# To the Editors of the Medical and Physical Journal

**Published:** 1802-03

**Authors:** H. Davies

**Affiliations:** Piccadilly


### *To the Editors of the Medical and Phyfical Journal.*

GENTLEMEN,

WHEN I fent two Cafes of Apoplexy for infertion in your ufeful Journal, it was with a profeffed defign not to enter into any thing Controverfial on the fubject then in debate. I took it for granted that other Correfpondents, of fuperior endowments, would be excited to communicate their fentiments on a difeafe fo generally formidable in its confequences to the human frame; and herein I was not difappointed in your laft Number. —Controverfial fubjects, however unpleafant in one point of view, are generally attended with this advantage, as they excite others to a clofer inveftigation of that which is *dubious* on the point, and not unfrequently to reflect light on the obfcurity in which they are involved; thereby aiding us more fuccefsfully to treat the variety of perplexing cafes which unavoidably fall to the lot of Medical Practitioners.

It is very defirable that fubjects of medical difpute fhould be managed without perfonal animofity, and as devoid of acrimony as poffible. This would evince that the Difputants were more emulous to ferve the general caufe, than to gratify their private feelings; which, on fuch occafions, are not always under the moft favourable influence. All will allow, that the Science, however improved, has not yet arrived at its *ne plus ultra*; there is, therefore, ample room for the exercife of candour and diffidence in treating of fubjects, particularly of the more ambiguous kind. Refpecting my obfervations on the exhibition of *Emetics* in cafes of Apoplexy, which Mr. Crowfoot faid, "might have been fpared," I did not wifh it to be underftood as applicable *particularly* to the Cafe then in difpute, but, in a general fenfe, as unfavourable where there are fymptoms of compreffion on the brain; in which fentiment I have the concurrence of moft practitioners; it ought not, therefore, to be mifconftrued into perfonal reflection: And as to Mr. C's pamphlet on the fubject, I doubt not but he has made ufeful remarks on the variety of appearances the difeafe in queftion puts on; and when it falls into my hands to perufe, it will be without prejudice, and, probably, with fome profit.

I am, &c.

